# Dual-Targeted Therapy Circumvents Non-Genetic Drug Resistance to Targeted Therapy

**DOI:** 10.3389/fonc.2022.859455

**Published:** 2022-04-27

**Authors:** Wei Wang, Yue Sun, Xiaobo Liu, Shaji K. Kumar, Fengyan Jin, Yun Dai

**Affiliations:** ^1^Laboratory of Cancer Precision Medicine, The First Hospital of Jilin University, Changchun, China; ^2^Division of Hematology, Mayo Clinic College of Medicine, Rochester, MN, United States; ^3^Department of Hematology, The First Hospital of Jilin University, Changchun, China

**Keywords:** targeted agent, non-genetic mechanism, dual-targeted therapy, parallel inhibition, drug resistance, cancer, linear inhibition, hematologic malignancy

## Abstract

The introduction of various targeted agents into the armamentarium of cancer treatment has revolutionized the standard care of patients with cancer. However, like conventional chemotherapy, drug resistance, either preexisting (primary or intrinsic resistance) or developed following treatment (secondary or acquired resistance), remains the Achilles heel of all targeted agents with no exception, *via* either genetic or non-genetic mechanisms. In the latter, emerging evidence supports the notion that intracellular signaling pathways for tumor cell survival act as a mutually interdependent network *via* extensive cross-talks and feedback loops. Thus, dysregulations of multiple signaling pathways usually join forces to drive oncogenesis, tumor progression, invasion, metastasis, and drug resistance, thereby providing a basis for so-called “bypass” mechanisms underlying non-genetic resistance in response to targeted agents. In this context, simultaneous interruption of two or more related targets or pathways (an approach called dual-targeted therapy, DTT), *via* either linear or parallel inhibition, is required to deal with such a form of drug resistance to targeted agents that specifically inhibit a single oncoprotein or oncogenic pathway. Together, while most types of tumor cells are often addicted to two or more targets or pathways or can switch their dependency between them, DTT targeting either intrinsically activated or drug-induced compensatory targets/pathways would efficiently overcome drug resistance caused by non-genetic events, with a great opportunity that those resistant cells might be particularly more vulnerable. In this review article, we discuss, with our experience, diverse mechanisms for non-genetic resistance to targeted agents and the rationales to circumvent them in the treatment of cancer, emphasizing hematologic malignancies.

## Introduction

Targeted therapy refers to the treatment specifically targeting a protein (oncoprotein in most cases) or dysregulated pathway that drives oncogenesis. Imatinib mesylate (Gleevec), a tyrosine kinase inhibitor (TKI) targeting BCR-ABL fusion oncoprotein for treating Ph+ chronic myelogenous leukemia (CML) (1), is considered as the first targeted agent for this approach. Another prototypic targeted agent is all-trans retinoic acid (ATRA), which acts to override the differentiation block mediated by PML-RARα fusion protein due to t(15;17) translocation in promyelocytic leukemia (PML) cells, resulting in the high efficacy of ATRA in treatment of PML (2). These successes have ignited enthusiasm to identify numerous novel molecular targets and develop a tremendous number of the first-in-class or best-in-class agents selectively against these targets. In consequence, we have witnessed an explosive increase in the number of targeted agents approved for the treatment of various cancer types, including both hematologic malignancies and solid tumors. The introduction of targeted therapy into the armamentarium for cancer treatment has initiated an era of precision medicine, which has been advanced with astonishing speed afterwards (3).

The notion of resistance to targeted agents is intimately associated with the concept of oncogene addiction (4), one of cancer hallmarks initially described in 2000 and subsequently updated and expanded (5–7). Although the mechanism by which oncogene addiction occurs remains to be elucidated with certainty, one concept holds that the genes responsible for malignant transformation may have certain lethal effects that must be overridden in order for transformed cells to survive (4). For example, c-Myc, a well-described oncogene that promotes cell proliferation, may exert a pro-apoptotic action in some circumstances. Under these conditions, over-expression of the anti-apoptotic gene Bcl-2 is required for survival of c-Myc-driven transformed cells (8). Then, such cells become dependent on Bcl-2 and thus susceptible to strategies targeting Bcl-2. In addition, transformed cells are equipped with powerful anti-stress properties to adapt not only intracellular stresses (e.g., oxidative, replicative, metabolic, etc.) during oncogenesis but also various extracellular insults (e.g., hypoxic, inflammatory, etc.) in tumor microenvironment, both of which must be overcome in order to preserve their survival and proliferative advantages over their normal counterparts (6). Moreover, oncogene addiction is dynamic due to clonal selection or evolution under therapeutic pressure, an event stemmed from tumor heterogeneity (9–11). It is common that new genetic alterations (e.g., point mutations) of either primary targeted oncogene or other related oncogenes occur during treatment with targeted agents, thus conferring resistance to those agents *via* such a genetic mechanism involving the change of addicting oncogene (named *de novo* mutation). Even more problematically, only a few types of cancer are addicted to only one oncogene for transformation and tumor cell survival, while the vast majority of malignancies rely on multiple alterations involving oncogenic and non-oncogenic proteins or pathways (12). Thus, the mechanisms of drug resistance are often multifaceted and highly heterogeneous at intratumoral or intercellular levels, as well as from genetic and non-genetic point of view (11, 13, 14). Of note, in addition to the well-recognized genetic mechanism, non-genetic mechanisms of drug resistance have been emerging as a much broader (not only for TKIs but also for non-TKI targeted agents) and more complicated challenge in cancer treatment.

In this review, we do not intend to provide a comprehensive overview of current understanding of overall mechanisms for resistance to targeted agents or strategies to circumvent them; a number of reviews dealing with these subjects have been published (9, 10, 12–15). Instead, we aim to focus on the non-genetic mechanisms by which tumor cells escape the lethal effects of targeted agents, and how rational strategies can be designed to solve this problem, with our experience.

## The Origin of Drug Resistance - Intrinsic Versus Acquired

**Figure 1:**
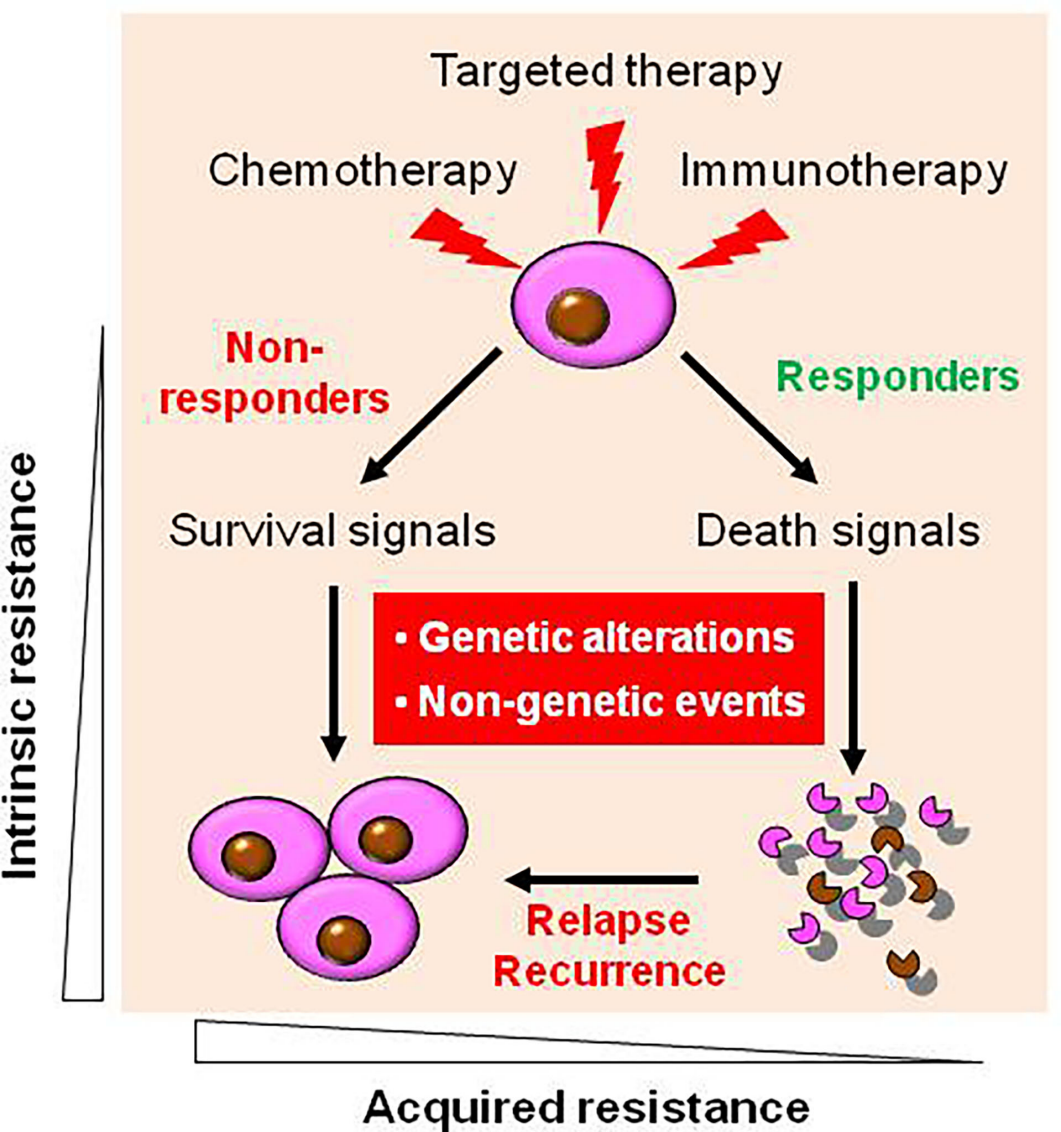
Diverse types of drug resistance to targeted therapy. During targeted therapy as well as conventional chemotherapy and novel immunotherapy, most patients carrying the driver genetic alterations respond to corresponding targeted agents, who are known as responders, while some patients who do not respond well, who are known as non-responder, due to intrinsic (primary) resistance. However, virtually all responders will eventually relapse and become resistant to agents targeting the original oncoproteins (as well as other targeted agents in most cases) due to acquired (secondary) resistance. Mechanistically, both intrinsic and acquired resistance stem from either genetic (e.g., *de novo* mutations) or non-genetic mechanisms, or both.

As in the case of more conventional chemotherapeutic agents, resistance to targeted agents may be either intrinsic or acquired (**Figure 1**) (16). During targeted therapy, most patients carrying the driver genetic alterations (e.g., EGFR mutations in non-small cell lung cancer (NSCLC) and fusion protein BCR/ABL in CML) would respond to corresponding targeted agents thus be benefited, while some patients do not respond well and thus are considered to experience primary or intrinsic resistance. However, only a few diseases like CML are addicted to single oncogene (e.g., BCR-ABL), most cancer types rely on multiple oncogenic alterations, with only partial dependency upon an individual target or pathway. Patients with NSCLC expressing certain activating EGFR mutations are much more likely to respond to EGFR inhibitors (17), and thus such tumors appear to be particularly addicted to EGFR signaling for survival. Unfortunately, some patients with NSCLC carrying EGFR mutations do not respond to EGFR inhibitors, suggesting EGFR mutation-independent mechanisms such as co-occurrence of KRAS mutations (18). Similarly, patients with colon cancer carrying KRAS mutations are unlikely to respond to TKIs directed against EGFR (19). A likely explanation for this phenomenon is that activation of the Ras/Raf/MEK/ERK pathway, which lies downstream of EGFR may bypass the addiction to EGFR activation. This may also apply to the case of the PTEN/PI3K/AKT/mTOR pathway, which is activated downstream of EGFR (20, 21). The development or pre-existence of PTEN mutations may, as in the case of mutant RAS, relieve transformed cells from their dependency on EGFR signaling. In this context, interventions capable of interrupting the PTEN/PI3K/AKT pathway (e.g., by PI3K inhibitors) have been shown to be effective in this setting (22). While the mechanisms underlying this phenomenon remain to be fully elucidated, one speculative possibility is that for reasons not yet understood, the activating mutations do not require or induce activation of “orthogonal” protective pathways (4). Alternatively, in the case of KRAS mutation that is commonly considered “undruggable” (11, 23), the activation of wild-type RAS by multiple receptor tyrosine kinases (RTKs) can confer resistance to mutated-KRAS (e.g., KRAS-G12C) inhibitors (in addition to *de novo* KRAS mutations) (24), suggesting a role of “horizontal” protective pathways (4). Consequently, inhibition of such activated compensatory signaling pathways in a linear or parallel manner may render interruption of the mutant RTK particularly lethal. More importantly, it should be kept in mind that although TKIs display significant activity in patients carrying oncogenic mutations, these targeted agents are not curative, and patients ultimately die of their disease. This raises a possibility that even in the case of susceptible disease with oncogenic mutations, interrupting complementary survival signaling pathways in combination with TKIs may improve patient outcome further.

Virtually all of patients who initially respond to targeted agents eventually develop acquired resistance to these agents, with no exception thus far. Such resistance may stem from *de novo* mutations in oncoprotein that prevent drug binding to their active sites (e.g., ATP-binding site in most cases) (25). The classic example is the development of point mutations (e.g., T315I or T790M) in the ATP binding pocket of BCR/ABL or EGFR, thereby conferring resistance to TKIs by preventing their binding to targets (26). Thus, *de novo* mutation represents a primary genetic mechanism for acquired TKI resistance (1). Furthermore, such new genetic alterations are not necessary to occur only in original targets, but also involve other oncoproteins (27). For example, acquired RET fusion proteins (e.g., CCDC6-RET fusion) in NSCLC cells bearing both primary and acquired EGFR mutations (e.g., del19 and L858R/T790M) confer resistance to both first- and second-generation TKIs (e.g., AZD9291/osimertinib) (18, 28). Similar phenomenon has also been observed in the case of acquired resistance to FGFR inhibitors in different cancer types bearing FGFR mutations or fusions (29). Both *de novo* FGFR gatekeeper mutations and activation of alternate RTKs account for acquired resistance to FGFR inhibitors. However, the distinction between acquired versus intrinsic resistance may be blurred in view of evidence that resistant cells carrying “*de novo*” mutations may pre-exist, while they remain dormant (like leukemic stem cells, LSCs) but expand after leukemic blasts carrying primary targets (e.g., BCR-ABL) are selectively eliminated by targeted therapy and eventually become dominant, a process known as clonal selection or evolution (11). A main strategy to overcome such mechanisms of drug resistance, either intrinsic or acquired, is to develop new-generation of TKIs active against mutant oncoproteins (26). However, although second- and third-generation TKIs are active to bind to and target these mutants, other *de novo* mutations (e.g., gatekeeper mutations such as T315I in BCR-ABL, which cannot be effectively targeted thus far) confer resistance to these next-generation TKIs again (26). Almost identical phenomena have been observed in the case of solid tumors, such as TKIs targeting activating EGFR mutations and EML4-ALK fusion protein in NSCLC (12). Another potentially promising strategy directed against either intrinsic or acquired mechanisms of resistance involves inhibition of critical pathways downstream of the original target (termed linear inhibition; see below). For example, because many kinases, including Aurora kinase A and Polo-like kinase 1 (PLK-1), operate downstream of BCR/ABL, inhibitors of Aurora kinases or PLK-1 may bypass the resistant barrier (e.g., T315I gatekeeper mutation of BCR/ABL) to induce apoptosis in imatinib mesylate-resistant cells (30–32). The advantage of such a strategy is that inhibiting such a downstream target or pathway eliminates the need to circumvent the primary resistance mechanism (e.g., *de novo* mutation), whatever its origin (intrinsic or acquired).

Similar mechanisms may also apply to immunotherapy such as monoclonal antibodies (MoAbs) targeting cell surface receptors like HER2 in breast cancer and CD20 in lymphoma. For example, in the case of MoAbs (e.g., the HER2 MoAb trastuzumab), resistance can be acquired *via* genetic alterations in the receptor (e.g., the presence of its mutant forms that do not bind to the MoAb), competition with endogenous ligands, activation of parallel or downstream pathways, or other immunological mechanisms (10, 33).

## The Nature of Resistance - Genetic vs Non-Genetic

Mechanistically, drug resistance to targeted agents, either intrinsic or acquired, can be divided into genetic (target-dependent) versus non-genetic (target-independent) (**Figure 1**) (34). In this classification, the former is primarily related to oncogene addiction, while the latter often reflects the ability of transformed cells to escape or adapt to the lethal actions of targeted agents due to acquisition of the perturbations that protect tumor cells from lethality of targeted agents, a “bypass” mechanism (12, 27). For the genetic mechanism, the development of *de novo* mutations that prevent binding of a targeted agent to its target of interest represents a primary resistance mechanism as discussed above (35). Other mechanisms also involve addiction to multiple targets/pathways and pharmacokinetic reasons preventing achievement of effective plasma concentrations (36). For the non-genetic mechanism, replacement or substitution of tumor cell dependency often involves the activation of a complementary pathway, an event capable of transmitting alternative signals sufficient to survive from the lethal consequences of interrupting the primary pathway by a targeted agent (10).

A typical example is that the lethal consequences of blocking the Ras/Raf/MEK/ERK pathway (e.g., by BRAF inhibitors) can be compromised by the activation of the PI3K/AKT pathway in tumor cells (20, 21). Alternatively, up-regulation of anti-apoptotic proteins or down-regulation of their pro-apoptotic counterparts can abrogate the lethality of various targeted agents (especially including most TKIs) (37, 38). In general, such a non-genetic form of resistance involves a fundamental change that makes neoplastic cells no longer dependent upon primary oncogenic signals, which originally drive transformation, for their survival. Thus, the strategies to overcome drug resistance *via* increasing the degree or duration of target inhibition by pharmacokinetic means (36) or developing more potent next-generation agents are most likely to fail in this circumstance. Since tumor cells has developed, in response to a targeted agent, such a non-genetic mechanism that makes them independent of their oncogenic drivers for survival, the identification of alternative targets responsible for or involved in this form of resistance and the development of fundamentally different approaches are required to prime resistant tumor cells for death.

In this context, dysregulation of the apoptosis-regulatory machinery mediated by the Bcl-2 family represents a universal non-genetic mechanism for drug resistance to targeted agents. It has been well documented that the Bcl-2 family of pro- and anti-apoptotic proteins is ultimately responsible for determining the fate of tumor cells. Anti-apoptotic proteins (e.g., Bcl-2, Bcl-xL, Mcl-1, and A1) are often multi-domain proteins that promote cell survival either directly by preserving mitochondrial integrity, or indirectly by binding to and blocking the activity of pro-apoptotic proteins (an event known as neutralization) (37, 38). The pro-apoptotic proteins include multi-domain (e.g., Bak and Bax) and BH3-only proteins (e.g., Bim, Bid, Bik, Bad, Puma, Noxa, and Hrk) (37). Based on their mechanisms of action, these pro-apoptotic proteins can be further subdivided into activator (e.g., Bim, Bid, and Puma), which directly triggers mitochondrial injury, and sensitizer (e.g., Bad), which antagonize the functions of anti-apoptotic proteins (39). The lethal actions of various targeted agents that disrupt oncogenic signaling pathways are considered to be integrated at the level of pro- and anti-apoptotic proteins (40). For example, the intracellular levels and disposition of Bim and Bad is regulated *via* their phosphorylation by multiple upstream kinases involving major signaling pathways, particularly Ras/Raf/MEK/ERK and PI3K/AKT/mTOR (20, 41). Thus, simultaneous interruption of these pathways results in accumulation of Bad and Bim in tumor cells and thus enhances lethality (42). On the other hand, co-administration of Bcl-2 inhibitors can circumvent resistance to targeted agents due to increased expression of anti-apoptotic proteins (43). An alternative strategy is to bypass the barrier of the intrinsic, mitochondrial apoptotic pathway (due to up-regulation of anti-apoptotic proteins or down-regulation of pro-apoptotic proteins) by triggering the extrinsic apoptotic cascade *via* up-regulating and activating death receptors (44–46). Moreover, in addition to apoptosis, multiple other forms of programmed cell death (PCD e.g., necroptosis, ferroptosis, pyroptosis, etc.) with almost entirely distinctive mechanisms have been identified (47, 48), which may provide much more choices to develop therapeutic approaches, particularly for enhancing apoptosis and overcoming the resistance to apoptosis based on these unique mechanisms.

Another common non-genetic mechanism is related to autophagy, a term literally meaning “self-eating”. Autophagy is a process in which cellular constituents are catabolized in the lysosome, which provides a source of energy to maintain critical cellular functions (49, 50). Autophagy thus functions as a cytoprotective mechanism to protect cells from environmental insults as well as anti-cancer treatment (particularly targeted therapy) (50). Under conditions in which autophagy protects cells from the lethal effects of targeted agents, co-administration of autophagy antagonists may dramatically increase the lethality of targeted agents (51). However, autophagy can also contribute to cell death under other circumstances. Instead of simply inhibiting autophagy, targeting the key step (e.g., cargo-loading mediated by SQSTM1/p62) leads to “inefficient” autophagy, which may more selectively kill malignant cells (52). While it could be a challenge to develop small molecule inhibitors for this kind of autophagy adaptor proteins, nanocarriers may represent an alternative and promising approach to deliver siRNA and shRNA specifically targeting molecular components that regulate autophagy (e.g., Beclin-1, LC3-II, ATGs, or even SQSTM1/p62) (53).

## The DTT Strategy to Overcome Resistance - Linear vs Parallel Inhibition

Despite the diversity for the nature of resistance, it is certain that strategies will have to be tailored specifically to the mechanism(s) responsible for resistance. For example, improving drug pharmacokinetics through optimizing drug doses or schedules as well as ameliorating drug design (36, 54), or developing next-generation agents capable of inhibiting mutants resistant to first-generation inhibitors (55), is capable of overcoming target-dependent (genetic) resistance, but most likely not going to work for target-independent (non-genetic) resistance. For the latter, emerging evidence supports that dual-targeted therapy (DTT), which is here defined as inhibition of two or more survival-related targets or signaling pathways, represents a promising strategy, for target-dependent and particularly target-independent forms of resistance (55). In general, inhibition of multiple targets *via* DTT includes at least two ways - parallel versus linear inhibition.

### Parallel Inhibition

DTT that simultaneously inhibits two or more complementary oncogenic pathways, which cooperate to maintain transformed cell survival or confer resistance (intrinsic or acquired), may be effective when targeting either single pathway is no longer capable of triggering cell death. This kind of DTT is considered a parallel inhibition approach to overcome resistance to primary targeted agents (56). A classic example of such an approach involves the Ras/Raf/MEK/ERK and PI3K/AKT/mTOR pathways (21), both of which prevent cell death by promoting phosphorylation, at different amino acid residues, and subsequent degradation of pro-death proteins (e.g., Bim and Bad). In this context, several studies have demonstrated that regimens combining PI3K or AKT inhibitors and MEK1/2 inhibitors potently induce cell death in both solid tumor and hematologic malignancies (20, 22, 40, 57). Another example is to combine the second-generation EGFR TKI osimertinib, which is active against the most important *de novo* mutation T790M that confers resistance to first-generation EGFR TKIs, with RET inhibition to overcome resistance to osimertinib due to acquired RET fusion in NSCLC (28). Similarly, simultaneous inhibition of canonical and non-canonical NF-κB pathways can also potentiate lethality in drug-sensitive and -resistant cells (58). Moreover, a DTT approach, known as dual targeting of epigenetic therapy that combines a DNMT inhibitor with a HDAC inhibitor to simultaneously target two epigenetic mechanisms (e.g., DNA methylation and histone acetylation, respectively), has already been used to treat several myeloid malignancies (59). This approach may be extended to include agents targeting other epigenetic mechanisms (e.g., histone methylation) as well (60). Notably, as these therapeutics primarily target epigenetic modifications of DNA or histones involving the transcription-regulatory machinery, they would theoretically influence numerous downstream targets with various functions and signaling pathways. Thus, they could be good candidates suitable for the development of the DTT regimens. However, the caution needs to be taken that they may also increase the incidence of adverse effects due to the diversity of their targets.

**Figure 2:**
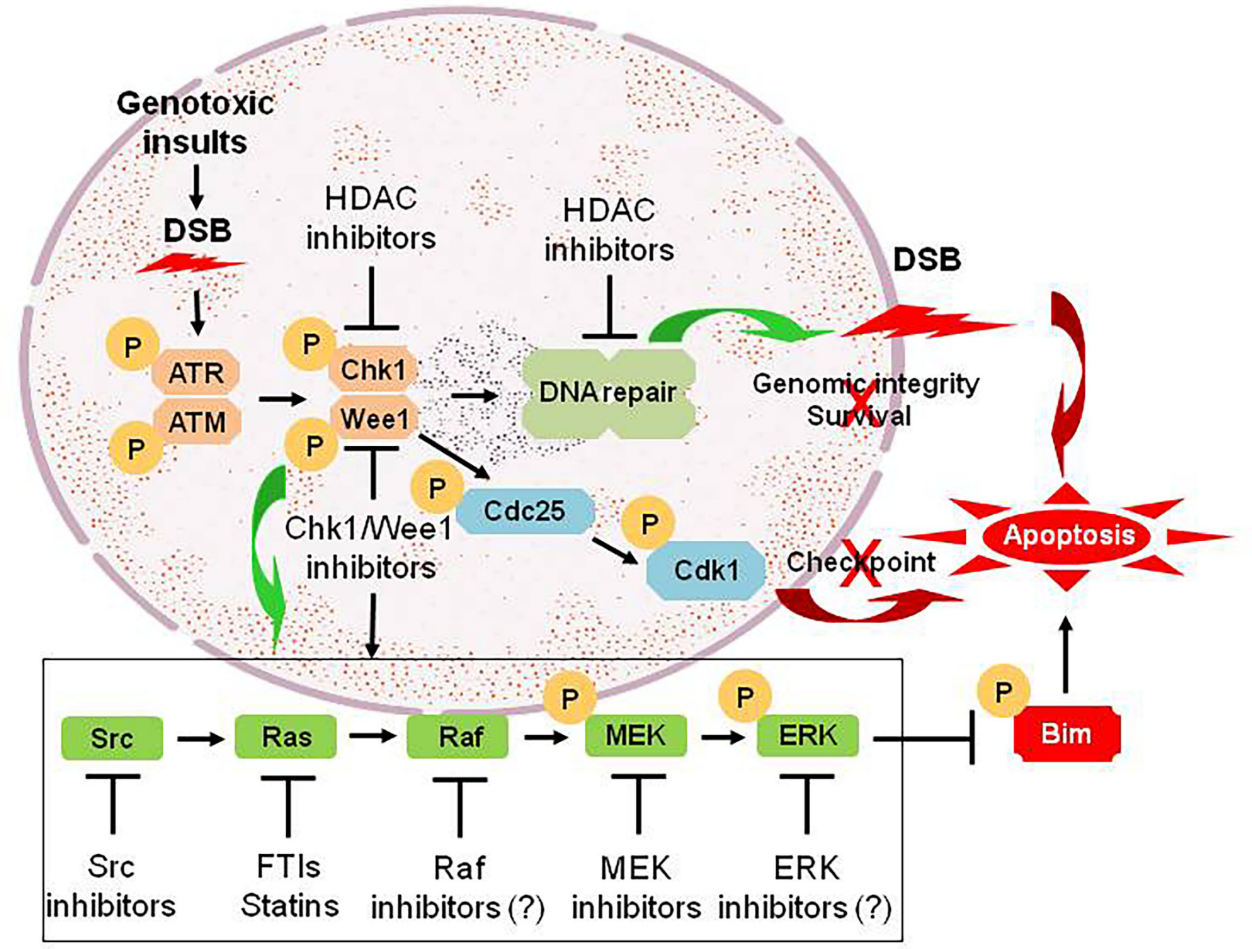
An example for the DTT approach *via* parallel inhibition. As DNA damage checkpoint and the Ras/Raf/MEK/ERK pathway represent two separate mechanisms for maintaining genomic integrity and survival of tumor cells under intracellular and extracellular stresses (e.g., genotoxic insults caused by conventional DNA-damaging chemotherapeutics). Treatment with Chk1 (or Wee1) inhibitors promotes DNA damage by abrogating checkpoints *via* Cdc25-mediated dephosphorylation of Cdk1 at inhibitory sites, an effect that could be potentiated by HDAC inhibitors *via* down-regulation of multiple genes involving DNA damage checkpoint and repair (linear inhibition). However, they also triggers activation of the Ras/Raf/MEK/ERK pathway *via* a not-yet-defined crosstalk between these two pathways, which most likely accounts for non-genetic resistance to Chk1 (or Wee1) inhibitors. Thus, a DTT approach *via* parallel inhibition of both DNA damage checkpoint (pathway #1) and its complementary Ras/Raf/MEK/ERK signaling cascade (pathway #2; e.g., by inhibitors of Src, Ras, Raf, MEK, and ERK, which act to prevent phosphorylation and degradation of pro-apoptotic proteins such as Bim, thus priming tumor cells for death induced by targeted agents like Chk1/Wee1 inhibitors), leads to unfixable DNA damage and thus triggers robust apoptosis. DSB, double-stranded break; P, phosphorylation.

Numerous links have been found between the cell cycle- and survival-regulatory machineries. For example, the Ras/Raf/MEK/ERK pathway has been implicated in the regulation of G2/M progression and in the disposition of cyclin D1, which is involved in the progression from G0/G1 into cell cycle (61). A DTT strategy targeting the cell cycle and survival signaling pathways involves inhibition of cell cycle checkpoints, most notably Chk1 or Wee1. Chk1 and Wee1 are key components of the DNA damage response (DDR), which trigger cell cycle arrest in cells subjected to genotoxic insults, allowing repair to occur if the damage is fixable, or apoptosis if it is not (62–64). This “self-checking” mechanism may be particularly important in maintaining survival of tumor cells harboring driver oncogenic mutations (64–66). Thus, Chk1 has been an attractive target for therapeutic intervention because it is involved in virtually all DNA damage checkpoints, and may also contribute to cell survival in a more direct manner (67). Most strategies involving the inhibitors of Chk1 or Wee1, as well as many other key components of various DDR pathways, have been combining them with various DNA-damaging agents (68). In this area, an elegant review article recently published has provided an overview of the advances and current status for the development of agents targeting DDR in various types of cancer (69). However, we observed that Chk1 inhibition triggers a compensatory activation of the Ras/Raf/MEK/ERK pathway in both hematologic malignancies and solid tumors, which may limit the lethal effect of Chk1 inhibitors (70, 71). Notably, abrogation of this signaling pathway at downstream sites (e.g., by MEK1/2 inhibitors) or more upstream sites (e.g., by farnesyltransferase or Src inhibitors) dramatically increases Chk1 inhibitor lethality (70–76). This phenomenon has been specifically attributed to potentiation of Chk1 inhibitor-mediated DNA damage (77), as well as up-regulation of the pro-apoptotic protein Bim, due to prevention of its phosphorylation and degradation *via* the ubiquitin-proteasome system (UPS) (78, 79). Such observations raise a possibility that in transformed cells, disruption of cell cycle checkpoints, which are often dysregulated in neoplasia, triggers a compensatory activation of the Ras/Raf/MEK/ERK pathway allowing them to survive, though the link between them remains unknown. Consequently, a DTT approach *via* parallel inhibition of these two critical survival pathways can lower the threshold for DNA damage-induced cell death (**Figure 2**), thus improving the anti-tumor activity of Chk1 inhibitors alone or in combination with conventional DNA-damaging agents (80, 81).

As a key family of anti-apoptotic proteins, Bcl-2 or its relatives (e.g., Bcl-xL and Mcl-1) are highly expressed in various types of cancer, particularly hematologic malignancies (e.g., lymphoma, leukemia, and multiple myeloma/MM), therefore representing one of the most attractive therapeutic targets (37, 38). However, it has taken a long time to develop the Bcl-2 inhibitor venetoclax (formerly ABT-199), which has been approved for the treatment of CLL and AML (82). One potential hurdle stems from a phenomenon that pro-apoptotic BH3-only proteins (e.g., Bim) released from one anti-apoptotic protein (e.g., Bcl-2) would bind to another anti-apoptotic protein (e.g., Mcl-1), thus disabling the lethal action of agents (e.g., Bcl-2 inhibitors) targeting only one arm of the apoptosis-regulatory machinery (83). Consequently, the activity of Bcl-2 inhibitors is inversely related to expression of Mcl-1 in tumor cells (83, 84). A corollary of this notion is that agents or interventions capable of down-regulating or inhibiting Mcl-1 could increase the activity of Bcl-2 inhibitors (85–87). Indeed, multiple such agents have been demonstrated to synergistically interact with Bcl-2 inhibitors in various hematologic malignancies. For example, CDK inhibitors that target transcription-regulatory CDKs (e.g., CDK9 and CDK7) and thus down-regulate Mcl-1 by disrupting the transcriptional regulatory apparatus (e.g., P-TEFb) *via* inhibiting the phosphorylation of the carboxy-terminal domain (CTD) of RNA Pol II (88–90). Analogous phenomenon has also found in the case of Mcl-1 down-regulation by B-Raf or MEK1/2 inhibitors (86, 91), or Bcl-xL down-regulation by PI3K/AKT inhibitors (92). An alternative approach is to up-regulate pro-apoptotic proteins (e.g., Bim) that prime tumor cells (e.g., by pre-occupying or saturating anti-apoptotic proteins) for death induced by Bcl-2 inhibitors (93). For example, HDAC inhibitors can up-regulate Bim in transformed cells (94), thus potentiating the activity of Bcl-2 inhibitors (95). Similarly, MEK1/2 and proteasome inhibitors prevent phosphorylation and following UPS-mediated degradation of Bim, thereby synergistically interacting with Bcl-2 inhibitors in hematologic malignancies (e.g., MM and lymphoma) (43, 96, 97).

Because the majority of targeted agents have multiple targets, attempts to understand the basis for interactions between them have been hindered by their complexity. Nevertheless, due to a variety of factors (e.g., the development of resistance or the presence or emergence of compensatory survival pathways), the need to interrupt two or more such pathways to achieve meaningful clinical benefits is now generally acknowledged. In addition, up-regulated expression of targeted oncogenic proteins also contributes to acquired resistance as observed in the case of mutant RTK-driven malignancies (1). Conventional strategies to circumvent this mechanism of acquired resistance include increasing drug doses, optimizing dosing schedules, or developing more potent next-generation kinase inhibitors active against mutant oncoproteins (55). However, these approaches may not be efficient enough, at least in certain circumstances, to overcome such a target-dependent mechanism of resistance. In this scenario, DTT combining targeted agents with inhibitors of other relevant targets/pathways *via* parallel inhibition provide an alternative, probably more effective, strategy to circumvent this resistance mechanism.

### Linear Inhibition

An alternative approach for overcoming resistance is to inhibit multiple targets involving two or more “orthogonal” pathways. In the other words, it attacks critical targets downstream of the primary target or its *de novo* mutant form, therefore circumventing target-independent (non-genetic) resistance (56). Such a DTT approach can be considered a “linear inhibition” strategy, which either improves the anti-tumor efficacy of targeted therapy (e.g., TKIs) or more importantly, overcomes its acquired resistance *via* a bypass mechanism. Linear inhibition often refers to blockade of a single pathway at two or more separate sites. For example, DTT can lower the threshold for cell death triggered by the primary targeted agent by inhibiting additional survival- or proliferation-regulatory pathways. In this case, dual inhibition of the driver oncogene and its downstream target (e.g., anti-apoptotic proteins of the Bcl-2 family), which is required for survival of tumor cells under oncogene-related stress (e.g., oxidative, replicative, metabolic, etc.) or in response to inhibition of the primary target, could yield a synergistic effect in both sensitive and resistant cells (98).

Clinical observations have shed light on the reciprocal nature of resistance versus sensitivity to targeted agents in a linear manner. For example, CML with overexpression and activation of other kinases (e.g., the Src family kinases such as Lyn, Hck, and Fyn) at downstream signaling cascade of BCR-ABL signaling are likely resistant to TKIs directed against BCR-ABL (99). Furthermore, these Src family kinases can phosphorylate BCR-ABL to alter its oncogenicity, a positive feedback to amplify this oncogenic signal. Thus, increased expression and activity of the downstream kinases of targeted oncoproteins may play an important role in determining the clinical response to TKIs and patient outcome. This may provide an explanation for the fact that multi-kinase inhibitors often display better activity against TKI-sensitive and -resistant tumor cells than those targeting only one kinase. For example, dasatinib, a dual-specific TKI targeting both BCR-ABL and the Src family kinases (e.g., Lyn) (26) is active against both imatinib-sensitive and -resistant CML *via* target-dependent or -independent mechanisms (99). In the latter, Lyn up-regulation and activation are associated with expression of Bcl-2, which is often silenced in BCR-ABL-positive CML cells, which in turn confers imatinib resistance (100). Thus, Bcl-2 inhibitors are able to overcome this form of imatinib resistance, suggesting a shift of oncogene addiction from BCR-ABL to Bcl-2 in these imatinib-resistant CML cells. Because Bcl-2 is a crucial survival factor for CML stem cells that are not addicted to BCR-ABL (26, 101), the most important factor for disease recurrence and TKI resistance (102), DTT targeting both RTK (e.g., BCR-ABL) and Bcl-2 thus represents a rational approach to circumvent acquired TKI resistance (100, 101). Other examples include simultaneous inhibition of PI3K and AKT or mTOR (103), inhibition of BCR/ABL and its downstream targets (e.g., Aurora kinase A and PLK-1) (30, 31), EGFR and MEK1/2 inhibitors (20), etc.

**Figure 3:**
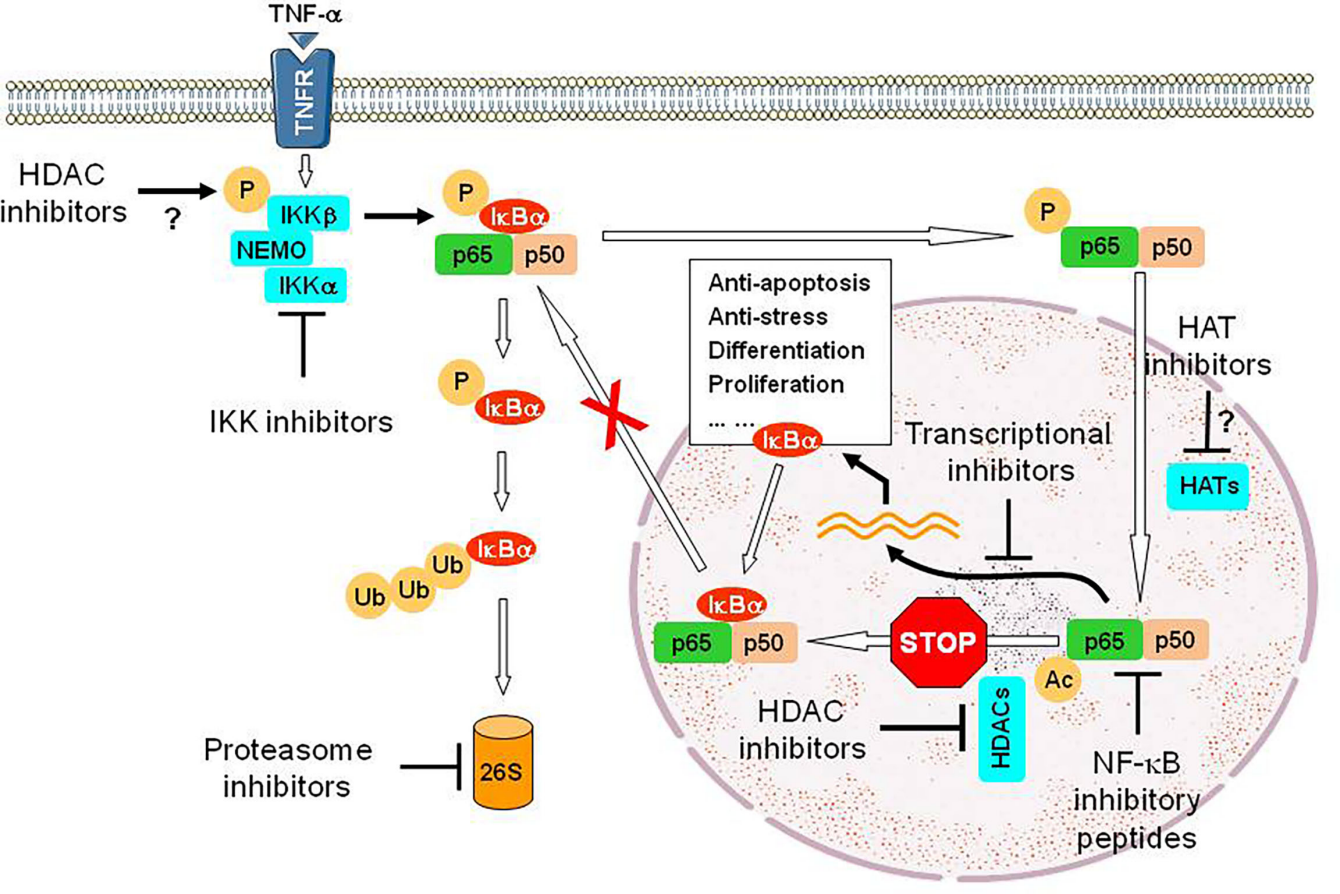
An example for the DTT approach *via* linear inhibition. While HDAC inhibitors exhibit anti-tumor activity *via* multiple mechanisms of action, exposure to HDAC inhibitors however activates the NF-κB pathway *via* post-translational modifications of RelA/p65, a major component of this critical survival pathway, including phosphorylation mediated by IKKβ (the mechanism for IKK activation by HDAC inhibitors remains unclear) and then acetylation mediated by HATs, but failure of its deacetylation due to inhibition of nuclear HDACs (e.g., HDAC1-3). Hyperacetylation of RelA/p65 prevents its nuclear export *via* binding of *de novo* synthesized IκBα, a downstream gene of NF-κB, resulting in sustained activation of NF-κB signal and therefore counteracting the lethal action of HDAC inhibitors. Thus, a DTT approach *via* linear inhibition of this non-genetic survival pathway at multiple sites can eliminate such an “off-target” effect of HDAC inhibitors and improve their efficacy as anti-tumor epigenetic therapy, though HDAC inhibitors often display limited single-agent activity. Disruption of these sites could involve IKK inhibitors that block phosphorylation of both IκBα and RelA/p65 (preventing IκBα degradation *via* the UPS and subsequent RelA/p65 entering into the nucleus), proteasome inhibitors that block proteasomal degradation of IκBα, (thus sequestering RelA/p65 in the cytoplasm), transcriptional inhibitors (e.g., inhibitors of CDK7 and CDK9) that block the expression of NF-κB-dependent genes), inhibitory peptides directly targeting RelA/p65 or its partner p50, and probably HAT inhibitors that block acetylation of RelA/p65. P, phosphorylation; Ub, ubiquitination; Ac, acetylation; 26S, 26S proteasome.

HDAC inhibitors, a class of epigenetic therapeutics, are truly pleiotropic agents that exert their anti-tumor activity through diverse mechanisms, including up-regulation of death receptors, generation of reactive oxygen species (ROS), disruption of multiple cell cycle checkpoints and DNA repair processes, down-regulation of survival-related proteins, and induction of pro-apoptotic proteins, among many others (104–106). Based on their multifaceted functions, it is not surprising that HDAC inhibitors have been shown to interact synergistically with multiple targeted agents as well as more conventional therapeutics, therefore representing an ideal candidate for the development of DTT (parallel or linear inhibition) (106, 107). A prototypical example for linear inhibition is activation of the NF-κB pathway as a compensatory response to HDAC inhibition. HDACs are responsible for deacetylation of multiple histones (primarily involving transcriptional regulation of gene expression) as well as numerous non-histone proteins involving cell cycle, DDR, DNA repair, cellular signaling, apoptosis, autophagy, RNA processing and stability, protein folding and aggregation, etc. (108). In this case, HDAC has also been named as lysine (K) deacetylase (KDAC). Among multiple proteins involved in cell survival decisions (105, 109), one such protein is RelA/p65, the most abundant component of the canonical NF-κB pathway, which plays an important role in drug resistance (acquired in particular) involving both solid tumor and hematologic malignancies (110, 111). Under basal conditions, RelA is bound by IκBα and sequestered in the cytoplasm, thus keeping the NF-κB pathway inactivated. Upon stimulation (e.g., by TNF-α), the activation of the IKK complex (consisting of IKKα/IKK1, IKKβ/IKK2, and IKKγ/NEMO) results in IKKβ phosphorylation (activation), which in turn phosphorylates IκBα and leads to its degradation *via* the UPS (112). This unleashes RelA, which then translocates into the nucleus where it is acetylated by histone (or lysine) acetyltransferases (HATs or KATs) and exerts its role as a transcription factor. RelA is then deacetylated by nuclear HDACs (e.g., HDAC1-3), an event required for binding of *de novo* synthesized IκBα and thus its nuclear export (113), as RelA itself lacks a nuclear export sequence. This process accounts for terminating NF-κB signal, thus making NF-κB activation as a short-term and reversible response in the case of TNF-α. However, hyperacetylation of RelA due to failure of its deacetylation (e.g., by HDAC inhibitors) leads to sustained NF-κB activation as observed in leukemic cells exposed to HDAC inhibitors (114), which in turn limits anti-tumor activity of HDAC inhibitors (115). Moreover, exposure to HDAC inhibitors also increases RelA phosphorylation (e.g., S365), an event mediated by IKKβ, which promotes its nuclear entry and susceptibility for acetylation by HATs (116). Notably, disruption of such compensatory NF-κB activation at either upstream (e.g., by IKK inhibitors that block phosphorylation of both IκBα and RelA) or downstream sites (e.g., by proteasome inhibitors that prevent IκBα degradation) interferes with RelA acetylation and nuclear import, resulting in down-regulation of NF-κB-dependent genes such as XIAP, cIAP1/2, Bcl-xL, and SOD2 (115–119). This intervention markedly increases the lethal action of HDAC inhibitors, suggesting another linear inhibition-based DTT approach (**Figure 3**). One of the examples for this approach is the combination of the HDAC inhibitor panobinostat and the proteasome inhibitor bortezomib, which has been approved to treat relapsed and refractory MM that are resistant to front-line therapy in virtually all cases. Similar phenomenon has also been observed in other hematologic malignancies (e.g., CLL and AML) when either pan-HDAC or class I HDAC inhibitors are utilized) (120, 121).

It is worth noting that DTT may be particularly appropriate when inhibition of a single component of the targeting pathway is incomplete and insufficient to trigger cell death. In this case, simultaneous interruption of this pathway at a second, downstream site may reduce survival signals below the threshold necessary to support survival. However, interruption of such a pathway at both upstream and downstream sites may be redundant, and could, at least theoretically, be counterproductive. For example, the lethal consequence of interruption of an upstream node may depend upon signaling imbalances stemming from activation of downstream targets. Thus, interruption of such conflicting signals could instead potentially attenuate the lethal consequences.

## Conclusion and Future Directions

To date, the bulk of evidence suggests that targeting a single oncogenic target or pathway would most likely be insufficient to achieve meaningful clinical responses and long-term survival of patients with most cancer types, despite a few exceptions (e.g., BCR/ABL inhibitors in CML and EGFR or ALK inhibitors in NSCLC). Diverse DTT approaches combining a targeted agent with another targeted agent, chemotherapy, or immunotherapy have been required for effective treatment and even cures of cancer (56), including hematologic malignancies such as diffuse large B-cell lymphoma (DLBCL), Hodgkin’s lymphoma, acute myeloid and lymphoid leukemia, and MM. Although it seems logic that targeted agents may have the capacity to enhance the activity of conventional cytotoxic agents, results with this strategy have not yet realized their potential. On the other hand, the rational combination of targeted agents, particularly those targeting complementary survival signaling or cell cycle regulatory pathways, in a parallel or linear inhibition manner, represents another promising DTT approach (**Table 1**). The notion of targeting two or more survival pathways specifically implicated in transformation offers the prospect of personalized therapy and the potential for therapeutic selectivity.

**Table 1:** Dual-targeted therapy (DDT) in hematologic malignancies and other cancers.

DDT strategy	Targeting pathway	MOA	Cancer type	Refs
Chk1/Wee1 inhibitor-based combinations				
Chk1 inhibitors + MEK inhibitors, FTIs, or Src inhibitors	DNA damage checkpoint & Ras/Raf/MEK/ERK pathway	Prevention of Bim phosphorylation and degradation; promotion of DNA damage; targeting myeloma stem cells; anti-angiogenesis; disrupting Ras farnesylation; activation of SEK1/JNK pathway	AML, MM, glioblastoma, breast, prostate	(70–78, 122–127)
Chk1 or Wee1 inhibitors + HDAC inhibitors	Epigenetic regulation & DNA damage checkpoints	DDR inhibition; disruption of DNA replication	AML	(63, 64)
Chk1 inhibitors + PARP1 inhibitors	DNA damage checkpoints & DNA repair	Potentiation of DNA damage	Breast, ovarian	(69, 128, 129)
HDAC inhibitor-based combinations				
HDAC inhibitors + DNMT inhibitors	DNA methylation & histone acetylation	Dual inhibition of HDACs and DNMTs; targeting CSCs	AML (approved), breast	(130, 131)
HDAC inhibitors + NAE inhibitors	DNA damage checkpoint & NEDD8	NF-κB inhibition; Bim up-regulation; inhibition of DNA repair	AML	(79)
HDAC inhibitors + TRAIL	Epigenetic regulation & extrinsic apoptotic cascade	Upregulation of DR4 and DR5	AML	(44, 132)
HDAC inhibitors +TKIs	Epigenetic regulation & oncogenic signaling	Disruption of chaperone function; overcoming TKI resistance	AML, CML, lung	(133–138)
HDAC inhibitors + Aurora kinase inhibitors	Epigenetic regulation & cell cycle	Potentiation of aurora kinase inhibition; overcoming TKI resistance	CML, kidney	(30, 139)
HDAC inhibitors + CDK inhibitors	Epigenetic regulation & cell cycle	Downregulation of Mcl-1 and p21^CIP1^ *via* inhibition of RNA Pol II	AML	(140, 141)
HDAC inhibitors + IKK inhibitors	Epigenetic regulation & NF-κB pathway	Prevention of NF-κB activation by blocking RelA acetylation	AML, MM	(115, 116)
HDAC inhibitors + Bcl-2 antagonists	Epigenetic regulation & apoptosis-regulatory pathway	Up-regulation and reactivation of Bim; autophagy inhibition	AML, MM	(51, 82, 95)
HDAC inhibitors + HSP90 antagonists	Epigenetic regulation & HSP90	p21^CIP1^ upregulation; Mcl-1 downregulation; inhibition of Bcr/Abl and its downstream STAT5	AML, CML	(142, 143)
HDAC inhibitors + IAP antagonists	Non-canonical NF-κB pathway & extrinsic apoptotic cascade	NF-κB inhibition; caspase 8 activation	MM	(46)
HDAC inhibitors + MLL-menin antagonists	DNA damage checkpoint & MLL-menin interaction	Disruption of DNA damage checkpoint and DNA repair	AML	(144)
Proteasome inhibitor-based combinations				
Proteasome inhibitors + HDAC inhibitors	UPS & epigenetic regulation	NF-κB inhibition; aggresome disruption; ER stress; Bim upregulation; ROS	MM (approved), CLL, ALL pancreatic cancer	(119–121, 145–148)
Proteasome inhibitors + CDK inhibitors	UPS & cell cycle	Bim upregulation; SAPK/JNK activation; NF-κB inhibition; Induction of ER stress	CML, AML, MM	(117, 149–151)
Proteasome inhibitors + Bcl-2 antagonists	UPS & apoptosis-regulatory pathway	Mcl-1 downregulation; SAPK/JNK activation; BAK activation; ROS	MM, MCL, DLBCL,CLL	(43, 96, 152, 153)
Proteasome inhibitors + IAP antagonist	UPS & cIAPs	Inhibition of canonical and non-canonical NF-κB pathways; Bcl-xL downregulation	MM	(58)
Proteasome inhibitors + XPO-1 inhibitor	UPS & NF-κB pathway	Nuclear localization of IκBα; overcome drug resistance	MM (approved)	(154, 155)
Bcl-2 antagonist-based combinations				
Bcl-2 antagonists + MEK inhibitors	Apoptosis-regulatory pathway & Ras/Raf/MEK/ERK pathway	Downregulation of Mcl-1	AML,	(86)
Bcl-2 antagonists + CDK inhibitors	Apoptosis- or autophagy-regulatory pathways & transcription-regulatory machinery	Mcl-1 downregulation by RNA Pol II inhibition; down-regulation of SQSTM1/p62 (inefficient autophagy); up-regulation of pro-apoptotic BH3-only proteins; BAK/BAX activation; ROS; JNK activation	AML, MM	(51, 85, 87, 89, 90, 97, 156)
Bcl-2 inhibitors + sorafenib	Apoptosis-regulatory pathways & oncogenic signaling	Mcl-1 downregulation; Bim upregulation	AML	(157)
Bcl-2 inhibitors + TKIs	Apoptosis-regulatory pathways & oncogenic signaling	Overcoming TKI resistance; Lyn inhibition; targeting CSCs	CML, Ph+ ALL	(100, 101, 158)
MEK inhibitor-based combinations				
MEK inhibitors + TKI	Ras/Raf/MEK/ERK pathway & Bcr/Abl	Co-inhibition of Bcr/Abl downstream signals	CML	(159)
MEK inhibitors + AKT/mTOR inhibitors	Ras/Raf/MEK/ERK pathway & P13K/AKT/mTOR pathway	Prevention of feedback ERK activation; prevention of BAD degradation; Bim upregulation	AML, Prostate cancer, breast cancer, melanoma, colon cancer, glioblastoma	(20, 22, 41, 57)
MEK inhibitors + proteasome inhibitors	Ras/Raf/MEK/ERK pathway & UPS	ERK inhibition; RANKL inhibition	MM	(160)
MEK inhibitors + sorafenib	Ras/Raf/MEK/ERK pathway	Bim upregulation; Mcl-1 downregulation	DLBCL	(161)

MOA, mechanism of action; DDR, DNA damage response; HDAC, histone deacetylase; DNMT, DNA methyltransferase; CDK, cyclin-dependent kinase; NAE, NEDD8 activating enzyme; TKI, tyrosine kinase inhibitor; CSC, cancer stem cell; AML, acute myeloid leukemia, CML, chronic myeloid leukemia; MM, multiple myeloma, MCL, mantle cell lymphoma; DLBCL, diffuse large B-cell lymphoma; CLL, chronic lymphocytic leukemia; ALL, acute lymphoblastic leukemia.

According to our and others’ experience, optimization of a DTT approach requires addressing a number of unanswered questions. Among them, a key question is whether a targeted agent should have single agent activity in a particular disease in order to be of benefit in a DTT regimen. It is conceivable, although not formally proven yet, that a targeted agent inactive alone may also be able to potentiate the activity of another targeted agent if it disables a critical compensatory pathway. For example, while the HDAC inhibitor panobinostat does not show single agent activity in MM, it however enhances the efficacy of the proteasome inhibitor bortezomib in this setting, a DTT regimen approved to treat relapsed MM. Another key question is what a role of targeted agents that disrupts so-called “orthogonal” pathways downstream of driver oncogenes should play in the DTT strategies. They may ameliorate the otherwise lethal effects of oxidative, proteotoxic, DNA damage-related and other forms of stress due to the activation of oncogenes such as RAS and c-Myc. Although such inhibitors may not be as specific as those directly targeting oncoproteins that drive transformation (e.g., BCR/ABL in CML, FLT3 mutations in AML, and EGFR mutation or EML4-ALK fusion protein in NSCLC), they may disrupt the mechanisms required for maintaining survival of transformed cells and thus play an important adjunctive role in various DTT approaches. Notably, unlike in the case of TKIs, the mechanisms for drug resistance (either intrinsic or acquired) to non-TKI agents (e.g., proteasome inhibitors and IMiDs, the frontline therapy in MM treatment) remain largely unclear, most likely involving diverse and even more complicated non-genetic mechanisms (162, 163), although multiple DTT regimens (with undefined mechanisms for synergism) have already be successfully used in clinical practice.

The bulk of attention in this area of DTT has focused on the simultaneous interruption of two complementary survival pathways to achieve enhanced efficacy of targeted therapy thus far. However, some of the successful DTT approaches have involved more than two agents e.g., R-CHOP in DLBCL and several triplet regimens in MM. In this case, emerging evidence suggests that simultaneous interruption of more than two pathways may be required for maximal cell killing of transformed cells, or in the other words, to reduce the size of minimal residue disease (MRD), a main cause for disease recurrence (15). A future paradigm for such a DTT approach may combine targeted agents with more than two separate but somehow complementary mechanisms of action involving both linear and parallel inhibition, such as an inhibitor directly targeting oncoprotein (e.g., TKI) in conjunction with an inhibitor targeting potential compensatory survival pathway (parallel inhibition) and an inhibitor of an “orthogonal” pathway (linear inhibition).

Last, a curative approach may ultimately depend upon the eradication of both tumor cells (e.g., leukemic blasts) and cancer stem cells (CSCs e.g., leukemia-initiating cells) (164). Notably, CSCs seem not to addict to the oncogenic target or pathway for transformation (e.g., BCR/ABL for CML blasts but not CSCs) (165) but depend upon their unique survival pathways (166, 167). Indeed, a DTT approach combining inhibitors of such pathways for CSC survival and maintenance with agents targeting oncoproteins directly implicated in oncogenesis may yield results superior to those obtained with either agent alone or may overcome both genetic and non-genetic resistance (164, 167). A logical extension of this DTT approach would be to incorporate inhibitors of CSC-related pathways into the multi-agent regimens targeting two or more pathways described above. It is also worth mentioning that although resistance (either intrinsic or acquired) to immunotherapy (particularly immune checkpoint inhibitors such as PD-1 and PD-L1 MoAbs) has its unique mechanisms (e.g., those related to immune response and its regulatory machineries) (168), the principle of diverse resistance mechanisms and DTT approaches discussed above may also be implicated in this novel type of “targeted” therapy. Non-genetic mechanisms may also contribute to resistance to novel forms of targeted agents (e.g., PROTACs that act to degrade, rather than inhibit, targeted proteins) (169). In addition, with recent applications of single-cell sequencing techniques, dissection of intratumoral heterogeneity has helped identify distinctive targets and pathways in different clusters (clones) of tumor cells within the same tumor (170, 171). On the one hand, this could explain why many agents targeting a single oncoprotein (even though it drives malignant transformation or oncogenesis) presumably existed in dominant clones are not sufficient enough to kill the meaningful number of tumor cells. On the other hand, it provides a great opportunity for developing the DTT approaches that target multiple oncoproteins or survival pathways existed in different clones to achieve maximal killing of tumor cells. Given the large number of agents capable of inhibiting numerous targets currently available, DTT (linear or parallel inhibition, or both) may offer a chance of achieving the best response and long-lasting remissions or even cures of some cancer types, especially hematologic malignancies, otherwise considered fatal. Future progress in this effort is awaited with considerable anticipation.

## Author Contributions

YD and FJ conceptualized, wrote, edited, and revised the manuscript. YD and WW gathered and analyzed the literatures, and prepared the figures. YS and XL contributed to literature search and collection. SKK contributed to writing, editing, and revising the manuscript. All authors contributed to the article and approved the submitted version.

## Funding

This work was supported by the National Natural Science Foundation of China (Nos. 81471165, 81670190, 81671108, 81670189, and 81870160), the Natural Science Foundation of the Jilin Province (Nos. 20190201042JC and 20190201163JC), Science and Technology Development Program of the Jilin Province (No. 20210509010RQ), and Interdisciplinary Integration and Innovation Project of JLU.

## Conflict of Interest

The authors declare that the research was conducted in the absence of any commercial or financial relationships that could be construed as a potential conflict of interest.

## Publisher’s Note

All claims expressed in this article are solely those of the authors and do not necessarily represent those of their affiliated organizations, or those of the publisher, the editors and the reviewers. Any product that may be evaluated in this article, or claim that may be made by its manufacturer, is not guaranteed or endorsed by the publisher.
